# Geneticin (G418) resistance and electroporation-mediated transformation of *Fusarium graminearum* and *F. culmorum*

**DOI:** 10.1080/13102818.2014.996978

**Published:** 2015-01-23

**Authors:** Emre Yörük, Gülruh Albayrak

**Affiliations:** ^a^ Programme of Molecular Biology and Genetics, Institute of Science, Istanbul University, 34134Vezneciler, Istanbul, Turkey; ^b^ Department of Molecular Biology and Genetics, Faculty of Science, Istanbul University, 34134Vezneciler, Istanbul, Turkey

**Keywords:** *Fusarium graminearum*, *Fusarium culmorum*, electroporation, geneticin (G418), transformation, pFA6-kanmx4, pEGFP75

## Abstract

*Fusarium graminearum* and *F. culmorum* are phytopathogenic species causing scab and root rot diseases in all small grain cereals worldwide including Turkey. In this study, resistance levels to geneticin (G418) of 14 *F. graminearum* and 24 *F. culmorum* isolates collected from cereals were determined. Fungal cultures were grown on potato dextrose agar medium supplemented with 0, 25, 50, 75 and 100 µg/mL of G418. Minimum inhibitory concentration was determined as 25 µg/mL. As a result, it was concluded that all isolates were highly sensitive to G418. Plasmid pFA6-kanmx4 containing geneticin resistance gene (*kanmx*) was introduced singly or co-electroporated with pEGFP75 plasmid, containing GFP gene, into fungal protoplast cultures obtained with lytic enzyme. Transformants were grown in media including 25 µg/mL G418. Transformation frequencies were 2.8 and 1.8 transformant per µg plasmid for *F. graminearum* and *F. culmorum* isolates, respectively. Transformation process was also confirmed by spectrofluorimetric assay. Relative fluorescence unit values in co-transformants were calculated as 1.87 ± 0.04 for *F. graminearum* and 2.26 ± 0.08 for *F. culmorum*. The results obtained from the study gave information about antibiotic resistance levels of two *Fusarium* species in Turkey. Moreover, it was shown that pFA6-kanmx4 plasmid was a suitable vector, which can be used in genetic manipulation studies of these two fungal species in particular suppression of endogenous and/or the expression of exogenous genes.

## Introduction

Several strategies have been used in agriculture, in order to defend against phytopathogenic *Fusarium* species.[1,2] Development of resistant crop varieties to pathogen is one of the most common strategies. However, the process takes a long time. Also, developed varieties could have unfavourable agronomic traits. Applying the fungicides against pathogens is another approach to be used. Unfortunately, pathogen specific fungicides are absent. Therefore, there are some efforts for developing *Fusarium* isolates sensitive to fungicides.[2,3] The most natural approach is to provide the antagonistic effects of microorganisms on control of *Fusarium*.[4,5] Also, genetic manipulation of pathogen has been carried out in order to fight it.[6,7] The antibiotic resistance levels of a particular fungus will be used in genetic manipulation studies and should be tested according to designed transformation vectors.

Limited number of antibiotics, including amoxicillin, cefazolin, chloramphenicol, moxifloxacin, tobramycin and geneticin and hygromycin B, has been tested in studies associated with genetic manipulation of *Fusarium* genus.[7,8] Among them, hygromycin B resistance is well characterized in *Fusarium*. But no information has been available with respect to geneticin: G418 antibiotic resistance. Geneticin is an aminoglycoside antibiotic which inhibits the elongation step of translation in both prokaryotic and eukaryotic organisms. Therefore, it is not suitable for a cure. So it has been used as a selection marker in molecular studies.[9,10]

Vector-based gene manipulation approaches including gene disruption/deletion, gene silencing and/or over-expression have been effectively used for transformation of fungi by exogenous genes.[6,11,12] In fungal transformation studies, a polyethylene glycol (PEG)-mediated gene transfer method applied to fungal protoplast cultures has been well examined and efficient strategies have been developed in some species of Ascomycota.[6,7,13–15] However, different strategies gave different results on their stability and efficiency of fungal transformation. Electroporation-mediated gene transfer to fungal protoplasts is an alternative strategy.[16] Plasmid vectors used for transformation should include at least marker genes, reporter gene and proper promoter and/or terminator regions and replication origins. Since selection of transformants is generally carried out by using antibiotics, plasmid vectors should carry antibiotic resistant genes. Hygromycin B is the most preferred antibiotic in the selection of transformants in Ascomycota. Therefore, most of plasmid vectors contain hygromycin B resistance gene (hygromycin B phosphotransferase; hph). Fungus susceptible to antibiotic might have an ability to eventually develop resistance to antibiotics overtime as well as in nature.[8] Hence, effectiveness of different antibiotics and their related marker genes should be examined in gene manipulation studies. Geneticin:G418 resistance gene has been isolated from *Micromonospora rhodorangea*.[ 17] The gene can be used in fungal transformation studies by ligation of different plasmid vector scaffold. G418 included in plasmid vectors such as pFA6-kanmx4 could be used.

Determination of resistance levels of 38 isolates belonging to *F. graminearum* and *F. culmorum* to geneticin:G418 antibiotic was the major aim of this study. Transformation of two isolates belonging to two species by plasmid vectors containing G418 resistance gene via electroporation was also purposed.

## Materials and methods

### Fungal isolates, media and G418 resistance

Totally 14 *F. graminearum* and 24 *F. culmorum* single spore isolates obtained from scabby barley, maize and wheat were used in the determination of antibiotic resistance whereas transformation was carried out with only one *F. graminearum* (4F) and one *F. culmorum* (20F) isolate in this study (Table 1). Fungal isolates were grown on potato dextrose agar (PDA) plates for 10 days at 25 °C. Liquid cultures were also grown on potato dextrose broth (PDB) for 10 days at 120 rpm and 25 °C. The level of Geneticin:G418 resistance of isolates were evaluated by addition of G418 with various concentrations at 0, 25, 50, 75 and 100 µg/mL to PDA and PDB medium. Growth was determined via measurement of dry weight of mycelia and observing condiospore production.

**Table 1.** Fungal isolates used in this study.

Isolate	Species	Host	Region	Isolate	Species	Host	Region
F5	*F. graminearum*	Wheat	Sakarya	F2	*F. culmorum*	Wheat	Marmara
F6	*F. graminearum*	Wheat	Sakarya	F3	*F. culmorum*	Wheat	Konya
F7	*F. graminearum*	Wheat	Sakarya	F4	*F. culmorum*	Wheat	Marmara
F8	*F. graminearum*	Wheat	Sakarya	F10	*F. culmorum*	Wheat	Bilecik
F9	*F. graminearum*	Wheat	Balıkesir	F12	*F. culmorum*	Wheat	Balıkesir
1F	*F. graminearum*	Wheat	Bolu	F14	*F. culmorum*	Wheat	Bilecik
2F	*F. graminearum*	Wheat	Çankırı	F15	*F. culmorum*	Wheat	Sinop
3F	*F. graminearum*	Maize	Samsun	F16	*F. culmorum*	Wheat	Konya
4F	*F. graminearum*	Barley	Bolu	F17	*F. culmorum*	Wheat	Konya
5F	*F. graminearum*	Maize	Samsun	F19	*F. culmorum*	Wheat	Konya
6F	*F. graminearum*	Maize	Samsun	F20	*F. culmorum*	Wheat	Bilecik
7F	*F. graminearum*	Maize	Samsun	F21	*F. culmorum*	Wheat	Uşak
14F	*F. graminearum*	Wheat	Kastamonu	F24	*F. culmorum*	Wheat	Konya
15F	*F. graminearum*	Wheat	Sakarya	8F	*F. culmorum*	Wheat	Ankara
17F	*F. culmorum*	Wheat	Ankara	9F	*F. culmorum*	Wheat	Isparta
18F	*F. culmorum*	Wheat	Eskişehir	10F	*F. culmorum*	Wheat	Samsun
19F	*F. culmorum*	Wheat	Eskişehir	11F	*F. culmorum*	Wheat	Çorum
20F	*F. culmorum*	Barley	Dinar	12F	*F. culmorum*	Wheat	Amasya
F1	*F. culmorum*	Wheat	Marmara	13F	*F. culmorum*	Wheat	Konya

### Protoplast preparation

Mycelia harvested from fungal cultures grown on PDB medium were used in protoplast production. Novozyme 234 (Novo Industries, Denmark), Driselase (Sigma, Germany) and lytic enzyme (Sigma, Germany) obtained from *Trichoderma* sp. were used in the elimination of the cell walls. Enzymes were dissolved in digestion buffer (10 mmol/L Na_2_HPO_4_, 1.2 Mg_2_SO_4_, pH 5.8). Driselase, novozyme and lytic enzyme were added to liquid cultures with final concentration of 20 mg/mL. Six hours after enzyme addition, cultures were filtrated through Whatman paper grade 1 (Sigma, Germany). Two-fold volume of STC buffer (20% sucrose, 25 mmol/L Tris-HCl, pH 7.5, 25mM CaCl_2_) was added to the filtrate and samples were centrifuged at 800 × *g* for 10 minutes. Protoplasts were precipitated and then suspended by addition of STC buffer and they were used immediately in fungal transformation or stored for longer times at –70 °C.[14,18] The number of protoplasts were calculated and then diluted at final concentrations of 1 × 10^5^ for transformation studies.

### Plasmid vectors

**Figure 1.**
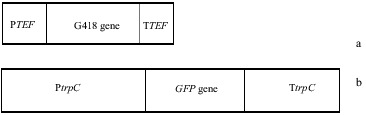
Diagrams of plasmid vector used in this study. (a) 1.3 kb fragment of pFA6-kanmx4 plasmid and (b) 2.6 kb fragment of pEGFP75 plasmids. PTEF: *A*. *gossypii* TEF promoter, TTEF: *A*. *gossypii* TEF terminater, Ptrp*C*: *A. nidulans* tryptophane promoter, Ttrp*C*: *A. nidulans* tryptophane terminator regions.

pFA6-kanmx4 and pEGFP75 plasmid vectors were used in this study. While pFA6-kanmx4 was provided from Dr Bedia Palabiyik, Department of Molecular Biology and Genetics, Faculty of Science, Istanbul University, pEGFP75 was a kind gift of Dr Hitoshi Nakayashiki from Plant Pathology and Microbiology Laboratory, Graduate School of Agricultural Sciences, Kobe University (Figure 1 A, B).

### Fungal transformation

Protoplasts at 1 × 10^5^ cells/mL and plasmid vectors (pFA6-kanmx4 and pEGFP75) were used in electroporation mediated transformation. Each plasmid was transferred into protoplasts as singly or multi. 390 µL of protoplast culture and 10 µL of plasmid (1 µg/µL) were mixed in sterile microtubes and samples were chilled on ice for 20 minutes. Samples were transferred to electroporation cuvette and electric pulse comprising of 800 V and 0.8 µs was carried out. Samples were immediately transferred to ice and kept for 20 minutes. Protoplast cultures were transformed with pFA6-kanmx4 plasmid and were diluted in molten medium with 1.2 mol/L sorbitol. After centrifugation at 2000 x *g* for 10 minutes protoplasts were inoculated to PDA medium including 25 µg/mL of G418. Transformation frequency was calculated as a number of transformants per µg plasmid DNA.[16,18–20]

### Selection of transformation

Confirmation of transformation was also carried out by co-introducing pFA6-kanmx4 with plasmid pEGFP75 [19] containing GFP gene, tryptophane promotor (Ptrp*C*) and terminator (Ttrp*C*). Co-transformation was detected by fluorescence microscopy (Leica-MX100, Germany) and spectrofluorometic measurements obtaining relative fluorescence unit values (RFU) at 485/535 nm excitation/emission wawelengths.[15,21] Chromosomal DNA was extracted from co-transformants by using commercial kit based on the establish CTAB protocol (Macherey–Nagel, Germany). PCR primers GFPF/GFPR (5′- ATGGTGAGCAAGGGCGAGGA-3′/5′- TTACTTGTACAGCTCGTCCA-3′) and GF/GR (5′-TGGGAAAAGAGAAGACACACG-3'/5′-GGACTGAACTCCCCTAGACA) targeting GFP and G418 resistance genes, respectively, were used in PCR analysis. PCR conditions were maintained as described by Wang et al. [14].

## Results and discussion

**Figure 2.**
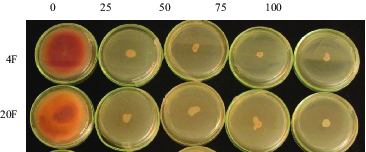
10-day-old cultures of (A) *F. graminearum* 4F and (B) *F. culmorum* (20F) isolates grown on PDA medium supplied with G418 at various concentrations as 0, 25, 50, 75 and 100 µg/ml.

In order to determine G418 antibiotic resistance levels of *Fusarium* species, isolates were maintained on PDA and PDB medium for 10 days. All control groups were grown on medium without antibiotic (Figure 2). However, none of fungal isolates were grown on medium supplemented with antibiotic at various concentrations as 25, 50, 75 and 100 µg/mL. Minimum inhibitory concentration (MIC) of G418 was thus set at 25 µg/mL in all *F. graminearum* and *F. culmorum* isolates.

**Figure 3.**
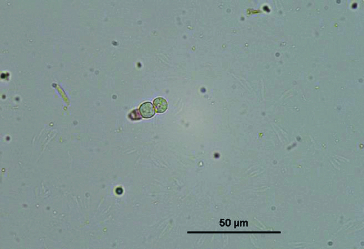
20X capture of protoplasts obtained from mycelia by incubation with lytic enzyme for 6 hours. Bar indicates 50 µm length.

Approximately, 5 mg fresh mycelia were used in protoplast preparation. Protoplast concentrations were calculated as 5 × 10^7^ cells/mL with lytic enzyme applications (Figure 3). However, 5 × 10^6^ protoplasts were obtained from cultures supplemented with Novozyme 234 and Driselase. It was shown that lytic enzyme was more effective than Novozyme 234 and Driselase for preparation of protoplast cultures.

**Figure 4.**
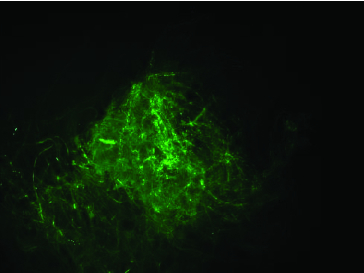
Fluorescence microscopy (20X capture) analysis of mycelia obtained from pEGFP75 transformed fungus.

**Figure 5.**
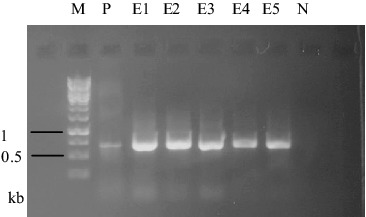
720 bp DNA bands amplified from 5 pEGFP75 transformed colonies and positive control (pEGFP75 plasmid). M: 1 kb DNA ladder, N: negative control, P: positive control, E1-6: transformant samples.

Fungal transformation was achieved by electroporation mediated-introduction of pFA6-kanmx4 plasmid including G418 resistance gene with *Ashbya gossypii* TEF promoter and terminator regions. Transformation was tested with protoplasts belonging to *F. graminearum* and *F. culmorum*. 1 × 10^5^ protoplasts of these fungal isolates were transformed with pFA6-kanmx4 plasmid. Isolates transformed with G418 resistance gene were efficiently grown on PDA + 25 µg/mL G418 medium. Transformation frequency was 2.8 and 1.8 transformant per µg plasmid for *F. graminearum* and *F. culmorum* isolates, respectively. For the confirmation of the process, co-transformation of pFA6-kanmx4 with pEGFP75 plasmid was carried out to protoplast cultures. Mycelia of co-transformants were investigated under fluorescence microscopy and mycelia with GFP fluorescence were detected (Figure 4). Moreover, RFU values in co-transformants were in the ranges of 1.87 ± 0.04–2.26 ± 0.08. GFP and G418 resistance gene fragments of 720 and 715 bp, respectively, were amplified from DNA molecules of transformants (Figure 5). These data showed that the co-transformation method was more successful than single transformation method in the two *Fusarium* species. Similarly, Wiebe et al. [18] and Scherm et al. [7] reported high levels (5 colony/µg DNA) of co-transformation efficiency in *Fusarium* species. Possible reasons of high transformation efficiency obtained of this study can be as follows: (1) efficiency of co-transformation depends on species and transformation conditions. (2) Introduction of genes carried by plasmid to related region occurs independently from transformation. (3) Also, insertion of one gene into a genome could mediate that of other genes.[22–24]

Geneticin:G418 resistance profiles and electroporation mediated gene transfer efficiencies of Turkish *Fusarium* isolates were investigated in this study. Results obtained from this study showed that all fungal isolates were highly sensitive to G418. Sakaguchi et al. [25] showed that *Trichosporon cutaneum* strains were at different hygromycin B-resistant levels. Similarly, *Penicillium roqueforti* and *Ophiostoma piceae* strains with different antibiotic resistance levels were reported by Durand et al. [19] and Wang et al. [14] Generally, hygromycin B has been selected as a marker gene in genetic transformation studies in Ascomycota. But, a great variation in the sensitivity to this antibiotic is present in fungi.[8] Thus, according to the output of this study, G418 could be used as an effective selective marker in molecular genetic studies.

In genetic transformation studies associated with Ascomycota, protoplast/spheroplast cultures have commonly been used as compared to spore cultures. In addition, PEG-mediated transformation is widely used. Wiebe et al. [18] reported that highest protoplast yield was obtained with Driselase application. In this study, protoplast cultures were obtained with three different agents and highest number of protoplast were obtained with lytic enzyme. Moreover, they obtained transformation efficiency in *F. graminearum* species at five transformants per µg DNA by PEG transformation. Higher levels of transformation efficiency were obtained by the electroporation-mediated gene transfer to protoplast cultures of *Fusarium* species in this study. Since transformation frequency in species belonging to Ascomycota showed high levels of variation ranging from 0.1 to 30 colony per μg DNA, different approaches for genetic transformation could be applied in fungal species. Co-transformation was also confirmed to be a stable and efficient transformation method in *Fusarium* species by electroporation. pAN7-1 containing hph gene or pEGFP75 plasmid with GFP gene are of approximately 6 kb sizes. The large sizes for plasmid could be problematic in fungal transformation.[21,26,27] Moreover, resistance to hygromycin B is variable in fungal species. Thus, plasmid pFA6-kanmx4 of 3.6 kb size could be useful in fungal transformation studies as a primary selectable marker since it has relatively small length. In this study, it was shown that toxicity of geneticin antibiotic was high for *Fusarium* species. Therefore, high level of MIC values was obtained even at the low antibiotic concentrations. For that reason, it was revealed that G418 gene carried by pFA6-kanmx4 could be useful as a selectable marker for transformation studies.

## Conclusion

This study reports on the geneticin resistance levels and the effectiveness of electroporation-mediated gene transfer of *Fusarium* isolates from Turkey. Results showed that fungal isolates were highly sensitive to this antibiotic. Additionally, plasmid vector including G418 gene was successfully transformed into protoplast cultures as singly and multi. The antibiotic resistance levels of *Fusarium* isolates which could be used in further gene silencing and/or over expression studies were tested. Findings of this study showed that geneticin antibiotic G418 could be utilized in practical applications of genetic transformation studies in *Fusarium* species.
